# Correlation between apoptosis and left ventricular remodeling in subacute phase of myocardial ischemia and reperfusion

**DOI:** 10.1186/s13550-015-0152-9

**Published:** 2015-12-10

**Authors:** Hiroshi Wakabayashi, Junichi Taki, Anri Inaki, Kazuhiro Shiba, Ichiro Matsunari, Seigo Kinuya

**Affiliations:** Department of Nuclear Medicine, Kanazawa University Hospital, 13-1 Takara-machi, Kanazawa, Ishikawa 920-8641 Japan; Division of Tracer Kinetics, Advanced Science Research Centre, Kanazawa University, 13-1 Takara-machi, Kanazawa, 920-8640 Japan; The Medical and Pharmacological Research Centre Foundation, Wo 32, Inoyama, Hakui, 925-0613 Japan

**Keywords:** ^99m^Tc-annexin-V, Apoptosis, Myocardial remodeling, Myocardial ischemia

## Abstract

**Background:**

To investigate whether an apoptotic process demonstrated by ^99m^Tc-annexin-V (^99m^Tc-AV) uptake correlates with left ventricular remodeling (LVR) after myocardial infarction, we assessed ^99m^Tc-AV uptake in rat model of myocardial ischemia and reperfusion.

**Methods:**

The left coronary artery (LCA) of 15 rats was occluded for 20 to 30 min, followed by reperfusion. After 2 weeks, ^99m^Tc-AV was injected, and then 1 h later, ^201^Tl was injected after reocclusion of the LCA. Dual-tracer autoradiography was performed to assess ^99m^Tc-AV uptake and the area at risk (AAR) by ^201^Tl defect. ^99m^Tc-AV uptake ratio was calculated by dividing the count density of the AAR by that of the normally perfused area. In short-axis LV slices, LV cavity dilation index (DI) was calculated by dividing the area of LV cavity by that of the whole LV area. LV wall-thinning ratio (WTR) was calculated by dividing the LV wall thickness in the AAR by that of the normally perfused area.

**Results:**

Significant ^99m^Tc-AV uptake in the AAR was observed in 10 rats. DI was significantly higher in rats with positive ^99m^Tc-AV uptake than in rats without uptake. WTR was smaller in rats with positive ^99m^Tc-AV uptake than in rats without uptake.

**Conclusions:**

The data suggest ^99m^Tc-AV uptake in injured myocardium might correlate with LVR at 2 weeks after myocardial ischemia and reperfusion.

## Background

Cardiomyocyte death by apoptosis during ischemia-reperfusion and myocardial infarction (MI) is associated with the progression of left ventricular (LV) dysfunction and remodeling [1–3]. The progressive loss of cardiomyocytes after acute MI (AMI) and ischemia-reperfusion may play a key role in the pathogenesis of heart failure.

Apoptosis is triggered through two main pathways: (1) the intrinsic pathway involves mitochondria and cytoplasmic reticulum, and (2) the extrinsic pathway utilizes cell surface receptor. Cellular stresses such as infarction and ischemia-reperfusion induce apoptosis through the intrinsic pathway [4–7]. These stimuli lead to the release of several factors into cytosol including cytochrome c, which activates the initiator caspase-9 via the apoptosome followed by the activation of effector caspases. The activation of downstream caspases leads to the cleavage of numerous structural and regulatory cellular proteins, thereby producing the apoptotic phenotype characterized by cell shrinkage, chromatin condensation, nucleus fragmentation, and externalization of phosphatidylserine (PS) on the outside of the cell membrane that serves as a signal for phagocytes. Annexin-V, a member of a phospholipid binding family of proteins, binds to PS-containing sites on the cell surface and has been suggested to be an early marker of apoptosis. Then, radiolabeled annexin-V has been used for the detection of apoptosis as a noninvasive imaging tool [8].

The process of myocardial tissue repair and healing after AMI is considered to consist of four phases based on the pathologic findings: cardiomyocyte death, acute inflammation, formation of granulation tissue, and scar formation [9]. After 2 weeks of MI, granulation tissue formation in the ischemic area is ongoing. The granulation tissue is still rich in inflammatory cells like lymphocytes and macrophages for helping to clear the cellular debris. At this point, the prolonged ^99m^Tc-annexin-V uptake by cardiomyocyte might correlate with a variety of tissue repair and healing.

Our previous studies showed that ^99m^Tc-annexin-V binding commenced at 0.5 h after ischemia-reperfusion in the mid-myocardium within the area at risk (AAR) and expanded to the subendocardial and subepicardial layers at 6 h after ischemia-reperfusion in rat models [10, 11]. The ^99m^Tc-annexin-V binding diminished gradually over 3 days and continued till 2 weeks after reperfusion. However, we could not assess the relation between ^99m^Tc-annexin-V uptake and LV remodeling, because we only had investigated five rats with 20 min of ischemia at 2 weeks after reperfusion.

Therefore, we investigated whether apoptosis or cell death demonstrated by ^99m^Tc-annexin-V uptake correlated with LV remodeling in rat models of myocardial ischemia-reperfusion at 2 weeks after reperfusion.

## Methods

### Animal model of acute ischemia and reperfusion

Male Wistar rats 8–9 weeks old were anesthetized with intraperitoneal administration of pentobarbital, 40 mg/kg, and were ventilated mechanically with room air. After left thoracotomy and exposure of the heart, a 7-0 polypropylene suture on a small curved needle was passed through the myocardium beneath the proximal portion of the left coronary artery (LCA), and both ends of the suture were passed through a small vinyl tube to make a snare. The suture material was pulled tightly against the vinyl tube to occlude the LCA. The occlusion time was 20 (*n* = 9) or 30 min (*n* = 6) for making differences in severity of ischemia [12, 13]. Myocardial ischemia was confirmed by ST-segment elevation on electrocardiography and by regional cyanosis of the myocardial surface. The snare was left loose on the surface of the heart for reocclusion of the LCA just before sacrificing the animals to identify the AAR.

At 2 weeks after reperfusion, ^99m^Tc-annexin-V (80–150 MBq) was injected via tail vein under anesthesia. After 1 h of the tracer injection, 0.74 MBq of ^201^Tl was injected just after reocclusion of the proximal portion of the LCA for delineation of the AAR. One minute later, the rat was euthanized by exsanguination in accordance with institutional guidelines and the heart was removed for analysis. The heart was rinsed in saline, embedded in methylcellulose, and cooled in a freezer. Serial short-axis heart sections of 20 μm thick were obtained using a cryostat to create a series of rings for autoradiography.

### Dual-tracer autoradiography

Dual-tracer autoradiography of the left ventricular short-axis slices was performed to assess ^99m^Tc-annexin-V uptake and AAR (^201^Tl uptake). The first exposure on an imaging plate (BAS-MS; Fuji Film) was performed for 15–20 min to visualize ^99m^Tc-annexin-V distribution 1–2 h after sacrifice. Three days later (12 half-lives of ^99m^Tc), the second exposure was made for 24 h to image the AAR expressed by ^201^Tl distribution.

All animal experimental protocols were approved by the Institute for Animal Studies of Kanazawa University.

### Radiolabeling of annexin-V

Mutant annexin-V (annexin V-117 mutant, a form of recombinant human annexin engineered to include a binding site for technetium) was prepared through expression in *Escherichia coli*. This material retains PS-binding activity equivalent to that of native annexin-V. A specific activity of 3.7–7.4 MBq (100–200 μCi)/μg of protein with a radiopurity of more than 90 % was achieved using a radiolabeling protocol [14].

### Data analysis

**Fig. 1:**
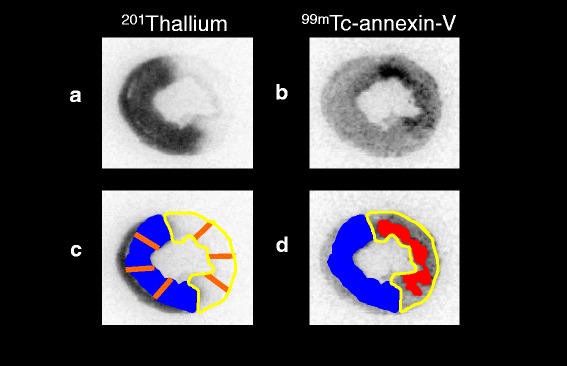
Measurement of parameters. The area at risk (AAR; *curved yellow line*) and normally perfused area (NA; *blue painting*) were defined from the ^201^Tl image (**a**, **c**), and these ROIs were applied to the ^99m^Tc-annexin-V images (**b**, **d**). Regions of interest were drawn manually over the area with easily identifiable ^99m^Tc-annexin-V uptake (**d**, *red painting*). The ^201^Tl image demonstrates the area at risk, while the ^99m^Tc-annexin-V image reflects the area and intensity of apoptosis. Each area’s wall thickness was calculated as an average of three radial lines (**c**, *orange line*) those equally divided the area into four

^99m^Tc-annexin-V accumulation was evaluated in three myocardial slices (20 μm thickness) at the mid-ventricular level spaced 1 mm apart from one another. Distribution of the tracers was determined by analysis of the digitized autoradiographs. The photostimulated luminescence in each pixel (100 × 100 μm) was determined using a bioimaging analyzer (BAS-5000; Fuji Film). For quantitative analysis, the uptake values for each region of interest (ROI) were expressed as the background-corrected photostimulated luminescence per unit area (1 mm^2^). A background ROI was set adjacent to the left ventricle. The AAR and normally perfused area were defined from the ^201^Tl image, and these ROIs were applied to the ^99m^Tc-annexin-V images to evaluate uptake of ^99m^Tc-annexin-V (Fig. 1). Regions of interest were drawn manually over the area with easily identifiable ^99m^Tc-annexin-V uptake. The ^99m^Tc-annexin-V uptake ratio was calculated by dividing the uptake value of the ^99m^Tc-annexin-V uptake area by that of the normally perfused area. All parameters obtained from the three slices in each rat were expressed as a mean value.

### Indexes of LV remodeling

In each three short-axis LV slices, LV cavity dilation index (DI) was calculated by dividing the area of LV cavity by that of the whole LV area. Then the average of DIs from three slices was considered as a representative value. LV wall-thinning ratio (WTR) was calculated by dividing the LV wall thickness in the AAR by that in the normally perfused LV area. Each area’s wall thickness was calculated as an average of three radial lines those equally divided the area into four. Then the average of WTRs from three slices was calculated as a representative value (Fig. 1).

### Histopathologic examinations with light microscopy

Hematoxylin- and eosin-stained (HE) slices adjacent to the slices used for autoradiography were examined histopathologically by light microscopy (×400).

### In situ detection of nuclear DNA fragmentation

Rat heart tissues were examined histopathologically for terminal deoxynucleotidyl transferase-mediated dUTP nick-end labeling (TUNEL) immunoreactivity to detect the presence of DNA fragmentation.

### Statistical analysis

For all statistical analysis, we used a statistical software package (JMP® SAS Institute Inc., Cary, NC, USA). Contingency table analysis was used for the relations between ischemic time and ^99m^Tc-annexin-V uptake. All results were expressed as mean value ± standard deviation (SD). Group comparisons were performed using analysis of variance. The Pearson product-moment correlation coefficient measures the strength of the linear relationship. A value of *P* < 0.05 was considered statistically significant.

## Results

The ratio of AAR to the whole LV area was not significantly different between 20 and 30 min of ischemia (mean value ± SD; 0.41 ± 0.05 vs. 0.53 ± 0.07, *P* = 0.2).

**Fig. 2:**
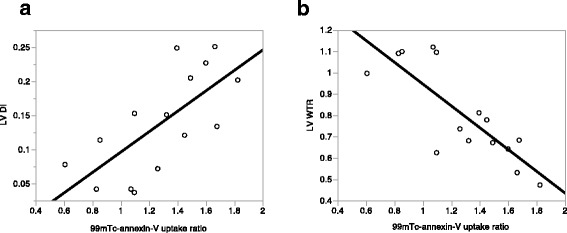
Comparison between the ^99m^Tc-annexin-V uptake and indexes of LV remodeling. ^99m^Tc-annexin-V uptake ratio correlated with LV DI [correlate coefficient = 0.70 (*P* = 0.0036)] (**a**) and WTR [correlate coefficient = −0.81 (*P* = 0.0002)] (**b**)

**Fig. 3:**
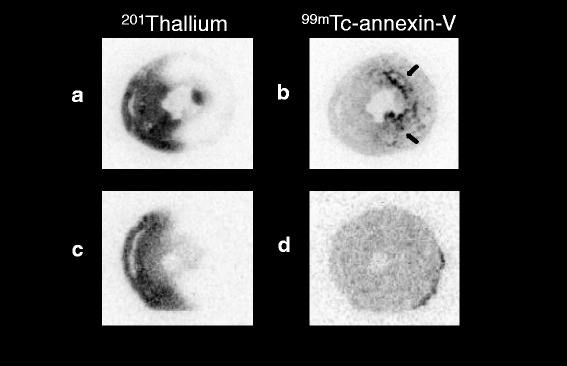
Autoradiography using of ^201^Tl and ^99m^Tc-annexin-V. After 30 min (**a**, **b**) and 20 min (**c**, **d**) of ischemia, ^99m^Tc-annexin-V was injected at 2 weeks. Single mid-ventricular slices are shown from representative animals. Significant ^99m^Tc-annexin-V uptake (*black arrow*) and morphological changes are observed in rat models with 30 min of ischemia

Significant ^99m^Tc-annexin-V uptake was (1) frequently documented in the AAR in 10 rats (66 % of all rats), especially in the case of a longer ischemia (100 % for 30 min of ischemia vs. 44 % for 20 min, *P* < 0.05) and (2) correlated with LV DI (mean value ± SD; 0.18 ± 0.06 vs. 0.06 ± 0.03, *P* = 0.0017) and LV WTR (mean value ± SD; 0.66 ± 0.10 vs. 1.08 ± 0.05, *P* < 0.0001), whereas it did not correlate with the ratio of AAR to the whole LV (mean value ± SD; 0.51 ± 0.04 vs. 0.41 ± 0.06, *P* = 0.1). Only two rats with 30 min of ischemia had ^99m^Tc-annexin-V uptake in both the AAR and normally perfused area adjacent to the AAR. LV DI and WTR were correlated with ^99m^Tc-annexin-V uptake ratio [correlate coefficient = 0.70 (*P* = 0.0036) and −0.81 (*P* = 0.0002), respectively] (Fig. 2). Representative cases were shown in Fig. 3.

**Fig. 4:**
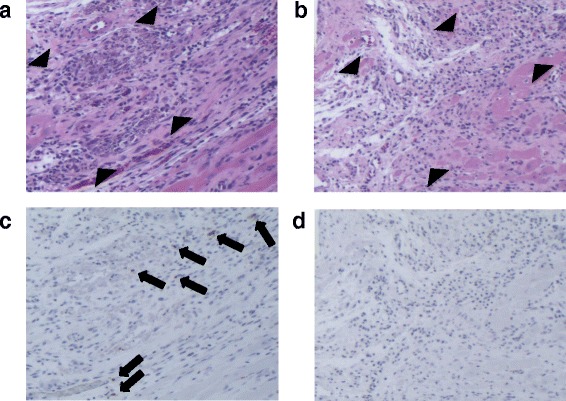
HE and TUNEL staining at 2 weeks after LCA occlusion and reperfusion. HE (**a**, **b**) and TUNEL (**c**, **d**) staining in the AAR in rats with (**a**, **c**) and without ^99m^Tc-annexin-V uptake (**b**, **d**) is presented. **a** and **c** as well as **b** and **d** are serial sections. Granulation tissue with inflammatory cells and fibrosis are seen widespread (*between black arrowheads*) (**a**, **b**). TUNEL-positive (*black arrow*) cells are detected in cardiomyocytes in rats with positive ^99m^Tc-annexin-V uptake (**c**). In rats with negative ^99m^Tc-annexin-V uptake, TUNEL-positive cells were rarely detected (**d**)

In the rats with 20 and 30 min of ischemia, light microscopic examination of the HE slices from frozen specimens showed that necrotic myocardium was replaced by granulation tissues with inflammatory cells. Scattered TUNEL-positive cells were detected in the AAR in rats with AV uptake, whereas TUNEL-positive cells were minimally observed in rats without ^99m^Tc-annexin-V uptake. Representative HE and TUNEL staining was shown in Fig. 4.

## Discussion

The present study confirmed that ^99m^Tc-annexin-V uptake was observed at 2 weeks after LCA occlusion and reperfusion in rats with a dilated LV cavity and thinned wall using autoradiography. Since ^99m^Tc-annexin-V uptake might reflect ongoing cell death of cardiomyocytes, the detection of apoptosis can be used as a diagnostic tool for predicting loss of cardiomyocytes in the vulnerable myocardial areas during the postinfarction recovery period.

^99m^Tc-annexin-V uptake seems to depend on the severity of ischemia-reperfusion injury in the rat model. Previous investigations demonstrated that apoptosis was observed at AAR after short-time ischemia and that apoptosis was involved extensively in border zone after long-time ischemia [15]. In this study, the size ratio of AAR to whole LV was not different between LCA occlusion time, but ^99m^Tc-annexin-V uptake in the AAR was significantly more observed in 30 min in the LCA occlusion model. Our results agreed with the previous findings that myocardial apoptosis appeared in ischemia-reperfusion area and that apoptosis was useful to evaluate ischemia-reperfusion damage.

Loss of myocardium caused by apoptosis in the acute phase of MI contributed to progressive myocardial dysfunction in human clinical studies. Human clinical studies demonstrated that increased uptake of ^99m^Tc-annexin-V was present in the infarct area in patients with AMI after percutaneous coronary intervention [16, 17]. In these studies, timing of monitoring apoptosis was based on the acute phase of MI, because apoptosis of myocardium was observed most strongly in the acute phase after ischemic damage.

Then, how long has apoptosis been observed in rat model? Palojoki et al. [18] showed that cardiomyocyte apoptosis occurred continuously over an extended period of time in the viable border zones of infarct scars in a rat model of permanent ligation. TUNEL-positive cardiomyocytes were numerous at 1 day after LCA ligation in both the viable border zones and the central infarct areas (average value 4.39 and 1.44 %). At later time points, scattered TUNEL-positive cardiomyocytes were observed in both the border zones adjacent to infarct scars and the remote myocardium (average value 0.34 and 0.09 % at 4 weeks, 0.10 and 0.04 % at 12 weeks, respectively).

Cardiomyocyte apoptosis occurs in advanced heart failure after the acute phase of MI. Olivetti et al. [19] demonstrated the existence of cardiomyocyte apoptosis morphologically and biochemically in patients who underwent cardiac transplantation for intractable congestive heart failure. Abbate et al. [20] examined an apoptotic rate (AR), the ratio of the number of cardiomyocytes co-expressing positive TUNEL and caspase-3 on nucleated cells per field, in both the infarct and the remote area in patients dying ≥10 days after AMI. The higher AR in the infarct area correlated with relative LV cavity dilation strongly but AR in the remote area did not. Apoptotic rate at the site of the infarction was increased nearly fourfold in patients with symptomatic heart failure at the time of initial hospitalization for AMI or subsequently before death versus remaining patients (median value 26.2 vs. 6.4 %). Our results consisted with their findings in that the positive ^99m^Tc-AV uptake in the AAR correlated with LV cavity dilation and thinned wall. Interestingly, the two rats demonstrated significant uptake expanded over the border zone between ischemic and normally perfused area after ischemia and reperfusion. Although apoptotic cardiomyocytes in remote non-infarcted areas contribute to the LV remodeling in rodent models of permanent ligation [18, 21, 22], such an apoptotic process over border zones in subacute phase had not been reported. In this respect, further study would be warranted to determine the significance of ^99m^Tc-AV uptake in the normally perfused area.

### Limitations

First, although ^99m^Tc-annexin-V uptake should depend on the externalized PS on apoptotic cells, the uptake also might reflect necrotic cells. There might be little binding of annexin-V to necrotic cells because myocytes have a significant intracellular content of (unlabeled) annexin-V. In rat cardiomyocytes, annexin-V is found predominantly on the sarcolemma and intercalated disks within the myocytes [23], and its content is extremely high (around 130 μg/g of wet weight) [24] and far greater than the concentration of the radiolabeled annexin-V, when 30 μg of labeled annexin-V are injected per rat (about 250 g of body weight) in our experiment. Based on the relative concentration gradient, exogenous radiolabeled annexin-V will not easily enter the intracellular environment and bind to PS competitively. Our study also demonstrated that the distribution of annexin-V was consistent with the area of TUNEL staining. Therefore, we believe that ^99m^Tc-annexin-V reflects apoptosis considerably if not completely. Second, ^99m^Tc-annexin-V uptake and morphological changes were evaluated only by autoradiography, because ^99m^Tc-annexin-V binding at 2 weeks was not expected to be high based on previous studies [10]. Although the uptake value was obtained semi-quantitatively by autoradiography, other imaging modalities including high-resolution animal SPECT, ultrasonography, and MRI may provide more accurate anatomical and functional information for monitoring serial change of ^99m^Tc-annexin-V uptake simultaneously.

## Conclusions

^99m^Tc-annexin-V uptake in injured myocardium correlated with LV remodeling at 2 weeks after myocardial ischemia-reperfusion. Ischemia-driven apoptosis or cell death demonstrated by ^99m^Tc-annexin-V uptake in the subacute phase might be a possible marker of LV remodeling.
